# Time is of the essence for auditory scene analysis

**DOI:** 10.7554/eLife.01136

**Published:** 2013-07-23

**Authors:** Andrew R Dykstra, Alexander Gutschalk

**Affiliations:** 1 **Andrew R Dykstra** is at the Auditory Cognition Lab, Department of Neurology, Ruprecht-Karls-Universität Heidelberg, Heidelberg, Germanyandrew.dykstra@med.uni-heidelberg.de; 2 **Alexander Gutschalk** is at the Auditory Cognition Lab, Department of Neurology, Ruprecht-Karls-Universität Heidelberg, Heidelberg, Germanyalexander.gutschalk@med.uni-heidelberg.de

**Keywords:** auditory scene analysis, temporal coherence, psychophysics, segregation, Human

## Abstract

Using computational models and stimuli that resemble natural acoustic signals, auditory scientists explore how we segregate competing streams of sound.

**Related research article** Teki S, Chait M, Kumar S, Shamma S, Griffiths TD. 2013. Segregation of complex acoustic scenes based on temporal coherence. *eLife* **2**:e00699. doi: 10.7554/eLife.00699

**Image** An acoustic stimulus in which elements of the target (black box) overlap in time and frequency with those of the background

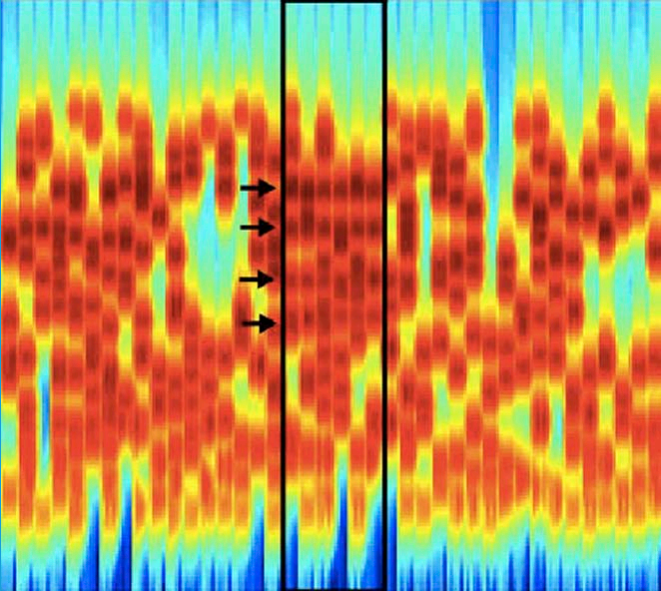

On a busy street corner or in a crowded bar, sounds from many different sources mix together before entering the ear canal. However, despite possessing just two ears, humans and other animals are remarkably adept at sorting out which sounds belong to which source. This process, known as auditory scene analysis (Bregman, 1990), is thought to underlie our ability to selectively listen to a single auditory ‘stream’ amidst competing streams: the so-called ‘cocktail party problem’ (Cherry, 1953; Broadbent, 1954). The loss of this ability is one of the most significant difficulties faced by individuals with hearing loss or damage to the auditory system, and may also be affected by the normal ageing process.

In contrast to the complexity of the acoustic environments we encounter on a daily basis, the vast majority of laboratory investigations into auditory scene analysis have used quite simple signals, often consisting of only a few elements (Figure 1A). Such stimuli have been used in an extensive body of research, including behavioural studies, neuroimaging experiments, and direct neuronal recordings. This research has told us a lot about the fundamental ways in which humans process sound, but some have questioned how relevant such simple stimuli are in understanding how we appreciate music or perceive speech at a cocktail party. Now, in *eLife*, Timothy Griffiths and co-workers—including Sundeep Teki and Maria Chait as joint first authors—report how they have used a new stimulus that more closely approximates natural acoustic signals to demonstrate that temporal coherence (that is, the coincidence of sound elements in and across time) is fundamental to auditory scene analysis in humans (Teki et al., 2013).

**Figure 1.**
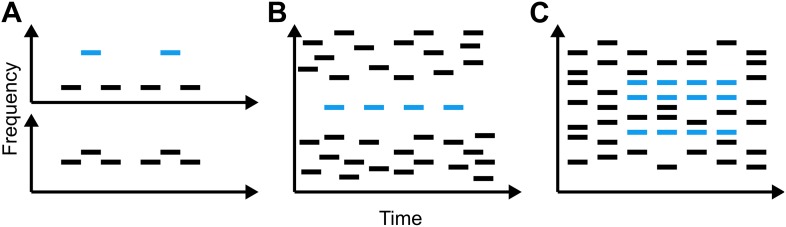
Representations of the relationship between time and frequency in three different types of stimuli that have been used to study auditory scene analysis. (**A**) The galloping ABA_ paradigm introduced by Van Noorden (1975): when subjects are played two tones that differ little in frequency (lower panel), they report hearing a single, galloping stream. Conversely, when the difference in frequency is large and the low and high tones are out of synch (upper panel), listeners report hearing two regular streams simultaneously. (**B**) The jittered informational masking paradigm introduced by Gutschalk et al. (2008): although the blue target tones are easy to discriminate visually from the multi-tone background, listeners do not always hear them. (**C**) The stochastic figure-ground stimuli introduced by Teki et al. (2011) (blue bars) contain elements of different frequencies, making them more like the sounds we encounter in everyday life than **A** and **B**.

The first models of our ability to segregate sound sources were based on data from behavioural, neurophysiological and imaging experiments in which subjects listened to various acoustic stimuli similar to those in Figure 1A and were asked to report whether they heard one or two streams of sound. The results of many such experiments are consistent with a model of auditory scene analysis in which the perception of a stream of sound is associated with the activity of a particular population of neurons, which can be readily distinguished from the activity of other populations (for a review see Micheyl et al., 2007). However, recent work has shown that sounds that clearly activate distinct neuronal populations can, when synchronous, result in the percept of a single stream (Elhilali et al., 2009). This led to the proposal that, subsequent to the auditory input being broken down into features such as pitch or spatial location, the sound from a single source is bound back together by temporal coherence between the neuronal populations representing its constituent features (Elhilali et al., 2009; Shamma et al., 2011).

Teki, Chait and co-workers—who are based at University College London, Newcastle University and the University of Maryland—extend previous work by devising a new ‘stochastic figure-ground’ stimulus (Figure 1C) that *requires* listeners to integrate information across time and frequency in order to perceive the blue ‘figure’ as separate from the background. They find that human listeners are quite sensitive to such figures. Furthermore, using computational modelling, they demonstrate that temporal coherence can at least qualitatively account for the results of behavioural experiments—which models based purely on the activation of separate populations struggle to explain. Because competing streams of speech also overlap in time and frequency, the data obtained with these stimuli further suggest that the brain could use this approach to solve the cocktail-party problem.

Although the current work is a substantial advance, and indicates that the human auditory system likely performs temporal coherence analysis, several questions remain unanswered. We know little about how or where this analysis might be performed in the brain, or how the results of such an analysis might be utilized by other brain regions. An earlier fMRI study revealed that activity in a region of the brain called the intraparietal sulcus increased when these new stimuli were perceived (Teki et al., 2011). They therefore propose that the intraparietal sulcus either carries out temporal coherence computations or represents their output. This leaves open the possibility that these stimuli, and auditory streams generally, are segregated at a relatively early stage of processing, perhaps in auditory cortex. This would be consistent with recent research using other types of stimuli (Figure 1B) (Gutschalk et al., 2008; Dykstra, 2011) as well as updated versions of the temporal-coherence model (Shamma et al., 2013).

Moreover, there are several phenomena that indicate that mechanisms other than, or in addition to, temporal coherence are required to fully explain how we perceptually organize sound. Bistable perception—whereby identical stimuli can give rise to two or more distinct percepts—is a particularly relevant example. On its own, temporal coherence cannot account for the fact that the same stimulus in the classical streaming paradigm (Figure 1A) can be heard as either one or two streams, or that the targets in an informational-masking stimulus (Figure 1B) are only sometimes perceived. The complex relationship between these sounds and the percepts they generate likely depends on additional mechanisms, acting both before and after the brain computes temporal coherence. However, this model provides a new framework within which to examine such questions, and should spark exciting new avenues of research in auditory scene analysis.
